# A functional role for Serum Amyloid A in the molecular regulation of autophagy in breast cancer

**DOI:** 10.3389/fonc.2022.1000925

**Published:** 2022-09-30

**Authors:** Manisha du Plessis, Tanja Andrea Davis, Daniel Wilhelm Olivier, Willem Johan Simon de Villiers, Anna-Mart Engelbrecht

**Affiliations:** ^1^ Department of Physiological Sciences, Stellenbosch University, Stellenbosch, South Africa; ^2^ Department of Internal Medicine, Faculty of Medicine and Health Sciences, Stellenbosch University, Cape Town, South Africa; ^3^ African Cancer Institute (ACI), Department of Global Health, Faculty of Medicine and Health Sciences, Stellenbosch University, Stellenbosch, South Africa

**Keywords:** autophagy, breast cancer, inflammation, cell signalling, serum amyloid A, proliferation

## Abstract

It has been established that the acute phase protein, Serum amyloid A (SAA), which is usually synthesized by the liver, is also synthesized by cancer cells and cancer-associated cells in the tumor microenvironment. SAA also activates modulators of autophagy, such as the PI3K/Akt and MAPK signaling pathways. However, the role of SAA in autophagy in breast cancer still remains to be elucidated. The aim of this study was to investigate the role of SAA in the regulation of signaling pathways and autophagy in *in vitro* and *in vivo* models of breast cancer. The MDA-MB-231 and MCF7 cell lines were transiently transfected to overexpress SAA1. A tumor-bearing SAA1/2 knockout mouse model was also utilized in this study. SAA1 overexpression activated ERK signaling in the MDA-MB-231 cells, downregulated the PI3K pathway protein, PKB/Akt, in the MCF7 cell line, while SAA1/2 knockout also inhibited Akt. Furthermore, SAA1 overexpression *in vitro* downregulated autophagy, while the expression of SQSTM1/p62 was increased in the MCF7 cells, and SAA1/2 knockout induced autophagy *in vivo*. SAA overexpression in the MDA-MB-231 and MCF7 cells resulted in an increase in cell viability and increased the expression of the proliferation marker, MCM2, in the MCF7 cells. Furthermore, knockout of SAA1/2 resulted in an altered inflammatory profile, evident in the decrease of plasma IL-1β, IL-6 and IL-10, while increasing the plasma levels of MCP-1 and TNF-α. Lastly, SAA1/2 knockout promoted resistance to apoptosis and necrosis through the regulation of autophagy. SAA thus regulates autophagy in breast cancer cells to promote tumorigenesis.

## Introduction

Tumor cells regulate the tumor microenvironment (TME) through the secretion of pro-inflammatory cytokines, which links tumor progression to inflammation (1). Infection, injury or changes in physiological tissue homeostasis elicits systemic responses such as the acute phase response to ensure the return to homeostasis. The acute phase response consists of a cascade of local or systemic reactions in response to inflammation, coordinated by cytokines, which are secreted by macrophages and other immune cells (2). Production of pro-inflammatory cytokines, mainly interleukins-1β (IL-1β) and -6 (IL-6) and tumor necrosis factor- α (TNF-α), at the site of injury stimulates the production of acute phase proteins (APPs) locally or by the liver. One such APP known to be synthesized primarily by the liver is serum amyloid A (SAA). SAA belongs to a family of evolutionary conserved proteins which is involved in inflammation (3, 4). The SAA isoforms are expressed at different levels in response to inflammatory stimuli. SAA1 and SAA2 are inducible APPs and share 96% nucleotide sequence homology. Human *SAA3* is considered a pseudogene, producing a non-coding RNA molecule, while *SAA4* is constitutively expressed (5). The murine SAA gene family consists of 5 variants, of which *Saa1*, *Saa2* and *Saa3* are all acute phase genes, *Saa5* is constitutively expressed and *Saa4* is a pseudogene (6). However, it has been reported that cancer and cancer-associated cells can also produce SAA in the TME, which adds a new dimension of complexity to the regulation of cancer by the TME (7). Elevated serum levels of SAA present in the TME are associated with poor prognosis. Additionally, various studies have shown that SAA contributes to cancer progression through the promotion of proliferation, metastasis, inflammation and angiogenesis in breast, lung, prostate, ovarian and renal cell cancers (4, 8–11). It has also been reported that SAA can activate the phosphatidylinositol-4,5-bisphosphate 3-kinase (PI3K)/Akt and mitogen-activated protein kinase (MAPK) pathway, including the MAPK components ERK1/2 and p38 (12–19). These pathways are known modulators of the intracellular degradation process, macroautophagy, hereafter referred to as autophagy (12, 20, 21).

Autophagy maintains homeostasis of non-malignant and cancer cells during metabolic and cellular stress. It is characterized by the sequestration of cytoplasm, long-lived proteins and cellular organelles in double-membrane vesicles called autophagosomes. These are delivered to and degraded in lysosomes, therefore this process is characterized as cellular self-consumption or self-eating. Defective autophagy induces metabolic stress which manifests as oxidative stress, endoplasmic reticulum stress, DNA damage and cytokine production, ultimately culminating in excessive inflammation (22). Autophagy can also regulate apoptosis and necrosis in solid tumors. As such, impaired autophagy is often associated with tumorigenesis (23).

It is clear from the literature that the pathogenesis of cancer is governed by multiple signaling pathways often induced by a pro-inflammatory TME. However, the complexity of the crosstalk between these pathways poses a challenge in understanding the role of SAA during cancer development. We identified a novel role for SAA, where it regulates the activation of intracellular signaling pathways and promotes cancer cell proliferation. Furthermore, our findings indicate that the overexpression of SAA1 promotes breast cancer progression by downregulating autophagy *in vitro*, while the double knockout of *Saa1* and *Saa2 in vivo* induces autophagy as a protective mechanism against cell death. In line with this, autophagy protected against apoptosis and necrosis *in vivo*, as well as inducing an altered plasma inflammatory profile in SAA1/2 double knockout mice.

## Materials and methods

### Cell lines and treatments

The human estrogen and progesterone receptor positive cell line, MCF7, and the triple negative, MDA-MB-231, human breast adenocarcinoma cell line were used in the *in vitro* study. The murine breast adenocarcinoma cell line, EO771, was used in the *in vivo* study. The MCF7 and the MDA-MB-231 cell lines were cultured in growth medium consisting of Dulbecco’s Modified Eagle Medium (DMEM) (41965062, Gibco^®^, ThermoFisher Scientific, MA, USA) supplemented with 10% fetal bovine serum (FBS) (FBS-GI-12A, Capricorn Scientific, Germany) and 1% Penicillin Streptomycin (15140122, ThermoFisher Scientific, MA, USA). The EO771 cell line was cultured in DMEM (Gibco^®^, ThermoFisher Scientific, MA, USA) supplemented with 10% FBS and 1% Penicillin Streptomycin. It was confirmed that cells were clear from mycoplasma infection. The cells were maintained in a humidified incubator set at 5% CO2 and 37°C. MCF7 and MDA-MB-231 cells were treated with the autophagy inhibitor, Bafilomycin A1 (Baf [B0025, LKT Laboratories]), 200 nM for 6 hours and 400 nM for 4 hours respectively. Autophagy was induced by using Rapamycin (Rapa [R0161, LKT Laboratories]), 50 nM for 24 hours in the MCF7 cell line, or serum starvation with 1% FBS for 8 hours in the MDA-MB-231 cell line.

### Transfections

The pcDNA3.1 (+) plasmid, kindly provided by Prof Sharon Prince (University of Cape Town, Cape Town, South Africa), was used as the control vector and was used as a backbone for the overexpression cDNA sequence for SAA1 (pcDNA3-hSAA1). The MCF7 and MDA-MB-231 cell lines were transfected at a confluency of approximately 80%. A total of 2 µg (6-well plate), 1 µg (12-well plate) or 0.3 µg (48-well plate) pDNA was transfected per well in a ratio of 1:3 with the X-tremeGENE™ HP DNA transfection reagent (6366236001, Roche, Sigma-Aldrich). The transfection complex was prepared in DMEM and the following volumes were added per well in the appropriate culture plates: 200 µl (6-well plate), 100 µl (12-well plate) or 30 µl (48-well plate). The pDNA:transfection reagent complexes were incubated for 30 minutes at room temperature (RT) and was added to the culture wells in a drop-wise manner. Transfection experiments were performed over 48 and 72 hours.

### WST-1 assay

The WST-1 Cell Proliferation Assay (11644807001, Roche, Sigma-Aldrich) measures cell viability based on mitochondrial reductive capacity. A 1:10 ratio of WST-1 (11644807001, Roche, Sigma-Aldrich) to volume per well was added to the growth medium. Following incubation at 37°C for 60 minutes, the colorimetric readings were measured at a wavelength of 450 nm with an EL800 universal microplate reader (BioTek Instruments Inc., VT, USA) and the KC Junior software (BioTek Instrument Inc., USA).

### Immunofluorescence

Cells were grown on sterile glass coverslips. Following transfections and autophagy modulation, the cells were fixed with 4% Paraformaldehyde (PFA) before being blocked in 5% goat serum for 1 hour at RT. The cells were incubated overnight at 4°C with anti-LC3-II primary antibody (1:200; 83506S; Cell Signalling Technology). The anti-mouse AlexaFluor-647 secondary antibody (1:500; A21236; ThermoFisher Scientific), prepared in PBS, was added to the cells, and incubated for 1 hour at RT. Cells were counterstained with 10 µg/ml Hoechst 33342 (14533, Sigma-Aldrich, MO, USA) for 10 minutes and mounted onto glass microscope slides with Dako Fluorescent Mounting Medium (Dako). Images were acquired on a LSM780 ELYRA PS1 confocal microscope (Carl Zeiss, Germany), using the Zeiss Zen Lite Black Edition Imaging Software v.2.3 (Carl Zeiss, Germany), as well as the Zeiss Axio Observer 7 fluorescent microscope (Carl Zeiss Microscopy, Munich, Germany). Images were acquired using the 63x oil immersion objective, using the same exposure times, and were captured in Z-stacks. The images were deconvoluted using the Icy software (24) and were analyzed using the Fiji^®^ (ImageJ) (Java 1.6_0 24 [64-bit]) software. The images were subjected to thresholding and quantified using the default ‘Analyze particles’ plugin with the Fiji^®^ (ImageJ) software.

### Animal model

Ethical clearance was obtained from Stellenbosch University Ethics Committee to assess tumor growth and the role of SAA in C57BL/6J wild-type (WT) mice and mice lacking the genes *Saa1* and *Saa2* (SAADKO, SAA Double Knock Out) (25) according to the approved protocol (ACU-2019-6426). Twenty-nine-week-old female SAADKO and 20 age-matched C57BL/6J control mice were used in this study. The syngeneic murine mammary carcinoma cell line, EO771, was used to inoculate breast tumors *in vivo*. A total of 1.5 x 10^6^/100 μl EO771 cells in Hanks Balanced Salt Solution (HBSS, Sigma Aldrich) were subcutaneously injected with 25-gauge needles into 9-week-old WT and SAADKO mice under 3% isoflurane (Isofor, Safeline Pharmaceuticals) sedation at the fourth mammary fat pad of each mouse. Control mice received 100 μl injections of HBSS at the same site and under the same conditions. Ten mice from the SAADKO and WT groups respectively were injected with 0.1mg/g chloroquine (CQ) dissolved in autoclaved water at the tumor site 6 hours prior to euthanasia. One half of the tumor was snap frozen in liquid nitrogen for use in western blot analysis. The other half was stored in formalin for use in hematoxylin and eosin staining. Furthermore, whole blood was collected into citrate tubes through means of cardiac puncture. The blood was centrifuged at 1000 x *g* for 10 minutes, using a Labnet Prism Microcentrifuge, to obtain plasma which was aliquoted and stored at -80°C for use in a milliplex assay. The tumor growth and survival curves are shown in **Supplementary Figure S1**.

### Western blot

Breast tumors were harvested, snap frozen in liquid nitrogen and stored at -80°C for use in western blot analysis. Tumor samples were cut into smaller pieces and incubated in modified radioimmunoprecipitation assay (RIPA) buffer with protease inhibitor cocktail. The samples were homogenized (Fisher Scientific) and were incubated on ice for 2 hours, where after the tumor lysates were centrifuged. The supernatant containing extracted protein from tumors and protein lysates from cell culture was quantified with a standard Bradford assay to determine total protein concentration, after which the samples were mixed with Laemmli’s sample buffer. Gel electrophoresis was performed to separate protein samples on 12% TGX FastCast gels (Bio-Rad, CA). Proteins were transferred onto polyvinylidene difluoride (PVDF) membranes and were blocked in 5% fat free milk, or in 5% bovine serum albumin for phosphorylated proteins, for 1 hour and subsequently incubated in primary antibodies overnight. The following primary antibodies were used: LC3-II (83506S; Cell Signalling Technology), SQSTM1/p62 (Sequestosome 1 [ab109012; Abcam]), MCM2 (ab108935; Abcam), Caspase 3 (ab184787; Abcam), PARP (ab191217; Abcam), phospho-ERK (4370; Cell Signalling Technology), total ERK (ab184699; Abcam), phospho-Akt (4060; Cell Signalling Technology), total Akt (9272; Cell Signalling Technology), phospho-p38 (9211; Cell Signalling Technology) and total p38 (9212; Cell Signalling Technology). The membranes were incubated in either anti-rabbit (7074S; Cell Signalling Technology) or anti-mouse secondary antibody (7076S; Cell Signalling Technology) for 60 minutes the following day. The membranes were developed with clarity ECL (Bio-Rad) and imaged on the ChemiDocTM MP System (Bio-Rad). Analyses were performed with Image Lab Software (Bio-Rad), using the total protein content of each membrane and a standard protein sample for normalization.

### Milliplex cytokine analyses

Plasma concentrations of the pro-inflammatory cytokines, IL-1β, IL-6, MCP-1 and TNF-α and the anti-inflammatory cytokine IL-10 was assessed by means of Milliplex assay. This assay was performed per manufacturer’s instructions and was detected by Luminex. The plasma samples were diluted with the kit constituent. Diluted samples were then loaded onto the Milliplex MAP Mouse cytokine/chemokine magnetic bead panel (MCYTOMAG-70K, Merck) plate, alongside standard curve samples. The beads were identified with flow cytometry and the results were quantified based on the florescent reporter signals.

### Histology, immunohistochemistry and image analysis

The formalin fixed tumor samples were processed using the Leica HistoCORE PEARL automated tissue processor, following a 20-hour pre-programmed time-schedule. The tumor samples were wax embedded using the Leica EG1150H tissue embedder. The samples were sectioned into 3 μm sections and were used for hematoxylin and eosin staining (H&E) to assess tissue morphology. Images were acquired on an ImageXpress^®^ Pico (Molecular Devices) and CellReporterXpress^®^ software (Molecular devices) and whole-slide acquisition was performed using the 4X objective.

### Statistical analyses

All data is represented as means ± SEM. Data was assessed for normality with the Shapiro-Wilk normality test. Statistical significance was determined with unpaired Student’s t-test, a one-way analysis of variance (ANOVA) with Bonferroni correction or a Kruskal-Wallis test. A value of p<0.05 was considered statistically significant. Analyses were performed with GraphPad Prism version 7 (San Diego, CA).

## Results

### The overexpression of SAA1 *in vitro*

**Figure 1:**
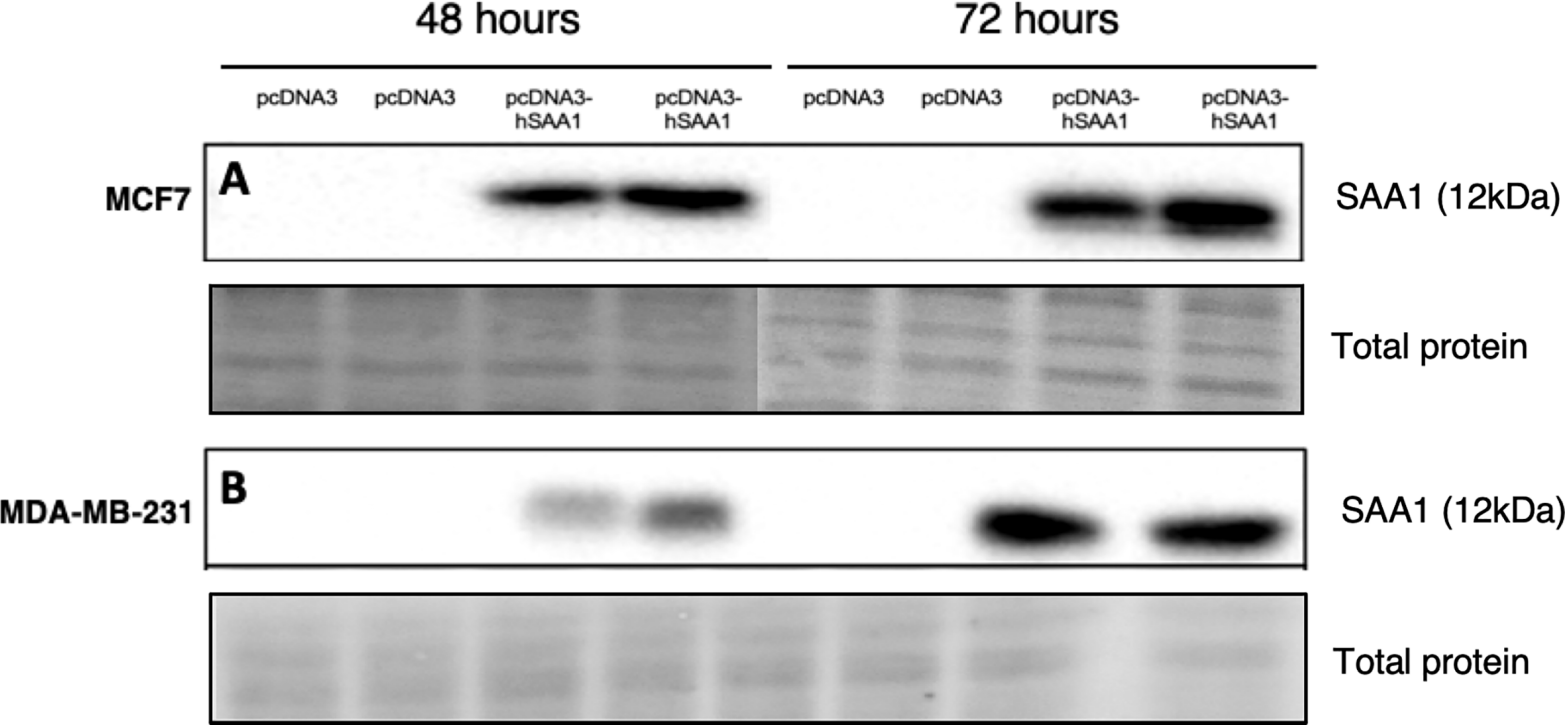
Representative western blot images confirming SAA1 overexpression *in vitro*. **(A)** SAA1 overexpression for 48 and 72 hours in the MCF7 cell line. **(B)** SAA1 overexpression for 48 and 72 hours in the MDA-MB-231 cell line.

The overexpression of SAA1 was confirmed with western blot analysis (**Figures 1A, B**), where little to no SAA1 expression was detected in cells transfected with the empty pcDNA3 control vector.

### SAA regulates MAPK and PI3K signaling pathway activation *in vitro* and *in vivo*

**Figure 2:**
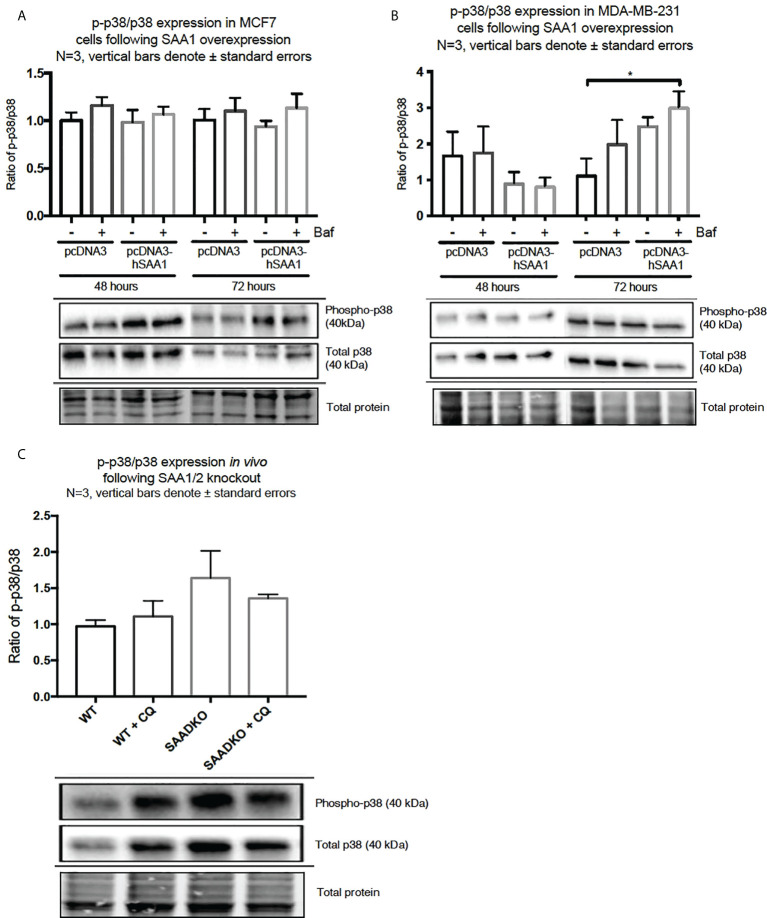
The activation of the p38 MAPK signaling pathway following SAA1 overexpression in human breast cancer cells, or in tumors from WT and SAADKO mice, as indicated by western blot analysis. **(A)** The activation of p38 following SAA1 overexpression for 48 and 72 hours in MCF7 cells. **(B)** The activation of p38 following SAA1 overexpression for 48 and 72 hours in MDA-MB-231 cells. **(C)** The activation of p38 *in vivo*. *p < 0.05.

**Figure 3:**
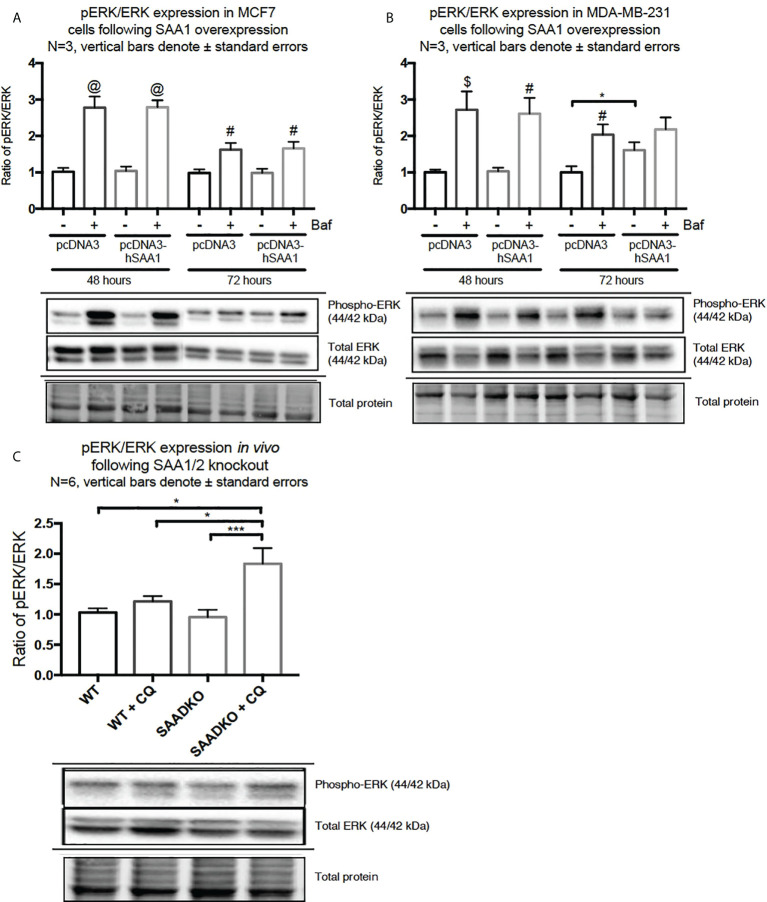
The activation of the ERK1/2 MAPK signaling pathway following SAA1 overexpression in human breast cancer cells, or in tumors from WT and SAADKO mice, as indicated by western blot analysis. **(A)** The activation of ERK1/2 following SAA1 overexpression for 48 and 72 hours in MCF7 cells. **(B)** The activation of ERK1/2 following SAA1 overexpression for 48 and 72 hours in MDA-MB-231 cells. **(C)** The activation of ERK1/2 *in vivo*. *p < 0.05, ***p < 0.001 when compared to the control groups. # Indicates p < 0.05, & indicates p < 0.01 and @ indicates p < 0. 0001 when comparing a group with its respective Baf treated group.

**Figure 4:**
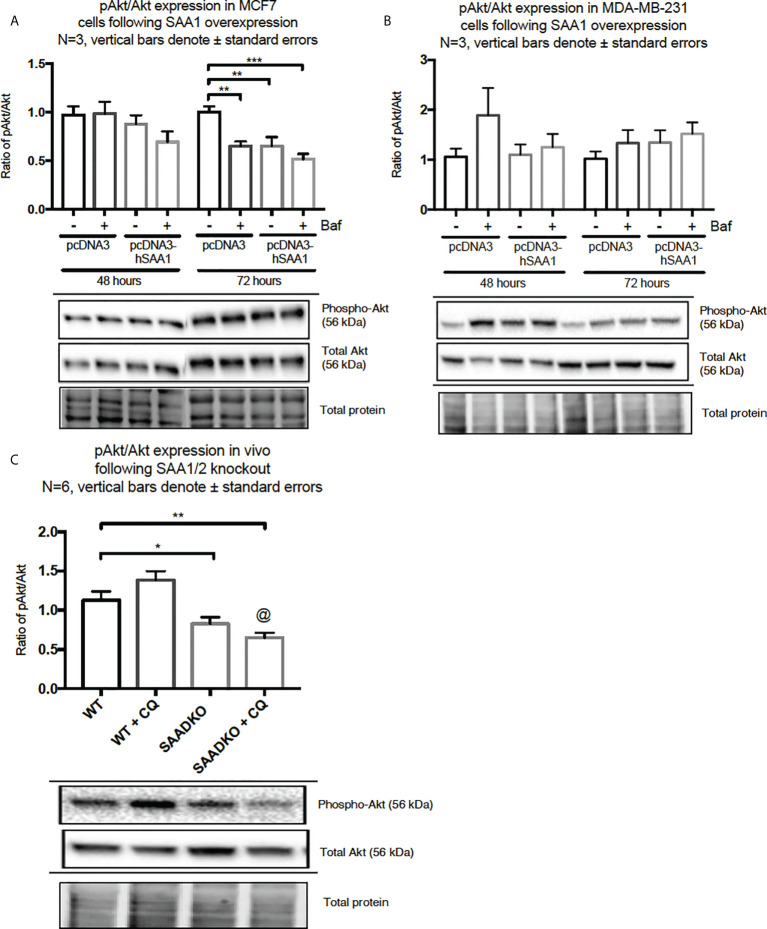
The activation of the Akt signaling pathway following SAA1 overexpression in human breast cancer cells, or in tumors from WT and SAADKO mice, as indicated by western blot analysis. **(A)** The activation of Akt/PKB following SAA overexpression for 48 and 72 hours in MCF7 cells. **(B)** The activation of Akt/PKB following SAA overexpression for 48 and 72 hours in MDA-MB-231 cells. **(C)** The activation of Akt/PKB *in vivo*. *p < 0.05, **p < 0.01, ***p < 0.001 when compared to the control groups. @ Indicates p < 0.0001 when comparing the WT+CQ and SAADKO+CQ groups.

The overexpression of SAA1 did not influence the activation of the MAPK, p38, in the MCF7 cell line (**Figure 2A**). However, SAA1 overexpression with autophagy inhibition increased the activation of p38 following 72 hours in the MDA-MB-231 cell line (**Figure 2B**). Furthermore, the knockout of SAA1/2 *in vivo* did not influence the activation of p38 in SAA1/2 double-knockout (SAADKO) tumors when compared to the wild-type (WT) tumors (**Figure 2C**). The overexpression of SAA1 increased the activation of ERK1/2 significantly in the triple-negative breast cancer cell line, MDA-MB-231, following 72-hour transfections (**Figure 3B**). No changes in the activation of ERK1/2 were observed in tumors from SAADKO mice (**Figure 3C**). However, the inhibition of autophagy significantly increased the activation of ERK1/2 when comparing to the SAADKO and WT tumor groups (**Figures 3A–C**). Interestingly, the overexpression of SAA1, as well as autophagy downregulation, significantly decreased the activation of Akt in the MCF7 cells. The knockout of SAA1/2 *in vivo* significantly decreased the activation of Akt when compared to the WT group. Lastly, the inhibition of autophagy in the SAADKO tumors significantly decreased the activation of Akt when compared to the WT control, however, this change was not significant when compared to the untreated SAADKO group (**Figures 4A–C**).

### Serum amyloid A regulates autophagy

**Figure 5:**
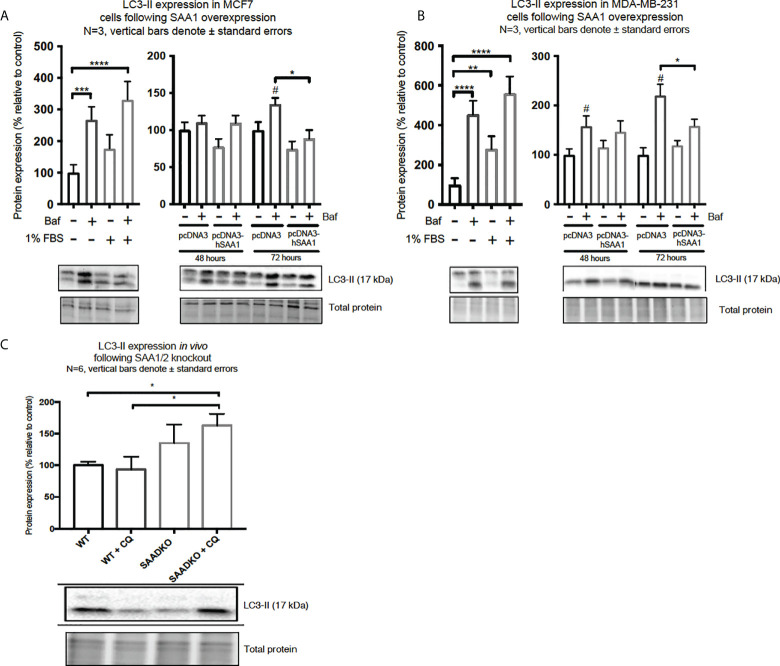
The expression of the autophagy marker, LC3-II, following SAA1 overexpression in human breast cancer cells, or in tumors from WT and SAADKO mice, as indicated by western blot analysis. **(A)** LC3-II expression following SAA1 overexpression for 48 and 72 hours in MCF7 cells. **(B)** LC3-II expression following SAA1 overexpression for 48 and 72 hours in MDA-MB-231 cells. **(C)** LC3-II expression *in vivo*. *p < 0.05, **p < 0.01, ***p < 0.001, ****p < 0.001 when compared to the control groups. # Indicates p < 0.05 when comparing a group with its respective Baf treated group.

**Figure 6:**
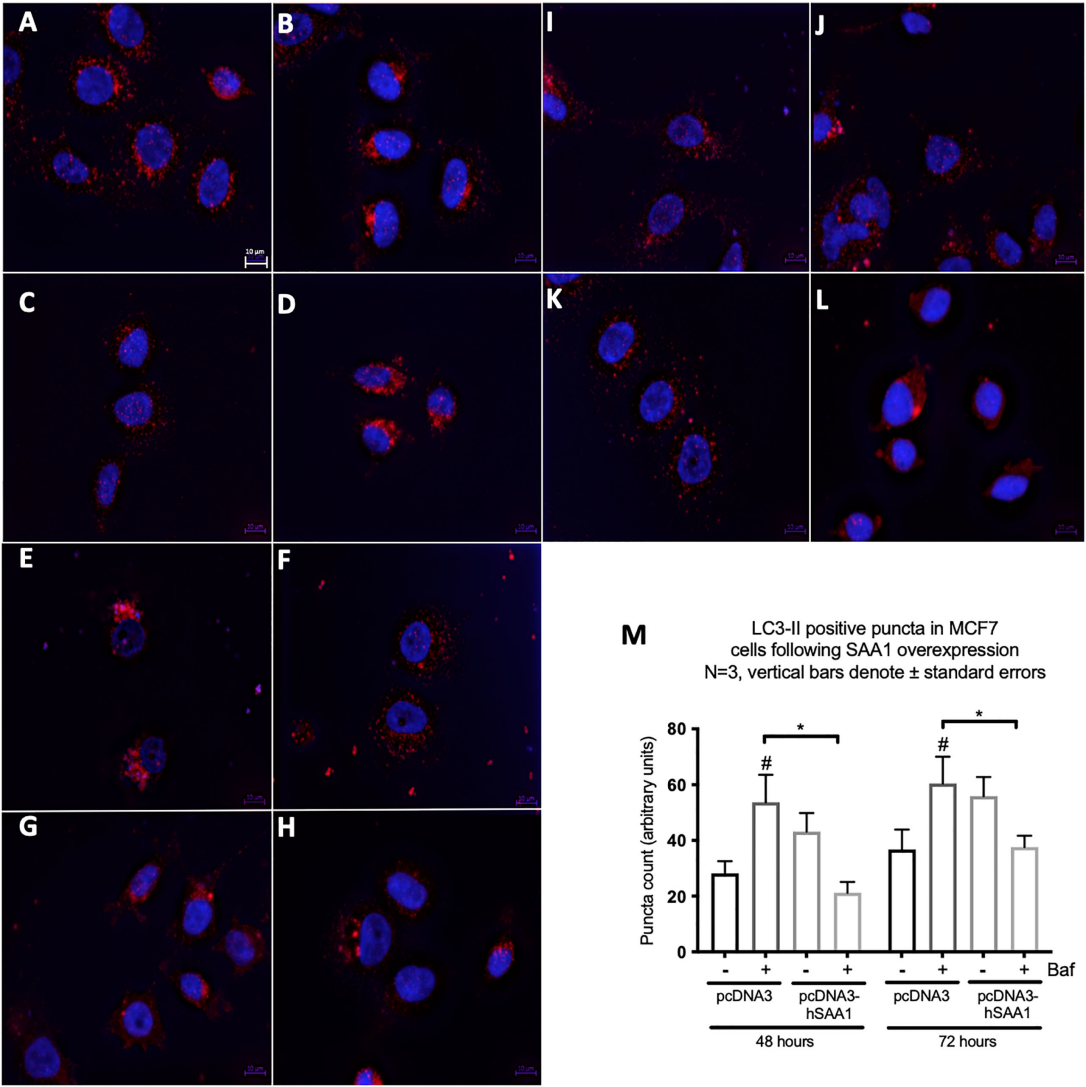
The expression of the autophagy marker, LC3-II, following SAA1 overexpression in the MCF7 cell line indicated by immunofluorescence and represented as mean fluorescent intensity. **(A)** Control. **(B)** 200 nM Bafilomycin for 6 hours. **(C)** 50 nM Rapamycin for 24 hours. **(D)** 200 nM Bafilomycin with 50 nM Rapamycin. **(E)** pcDNA for 48 hours. **(F)** pcDNA with Bafilomycin for 48 hours. **(G)** SAA1 overexpression for 48 hours. **(H)** SAA1 overexpression with Bafilomycin for 48 hours. **(I)** pcDNA3 for 72 hours. **(J)** pcDNA3 with Bafilomycin for 72 hours. **(K)** SAA1 overexpression for 72 hours. **(L)** SAA1 overexpression with Bafilomycin for 72 hours. **(M)** Mean fluorescent intensity of LC3-II following SAA overexpression for 48 and 72 hours. *p < 0.05, # Indicates p < 0.05 when comparing a group with its respective Baf treated group.

**Figure 7:**
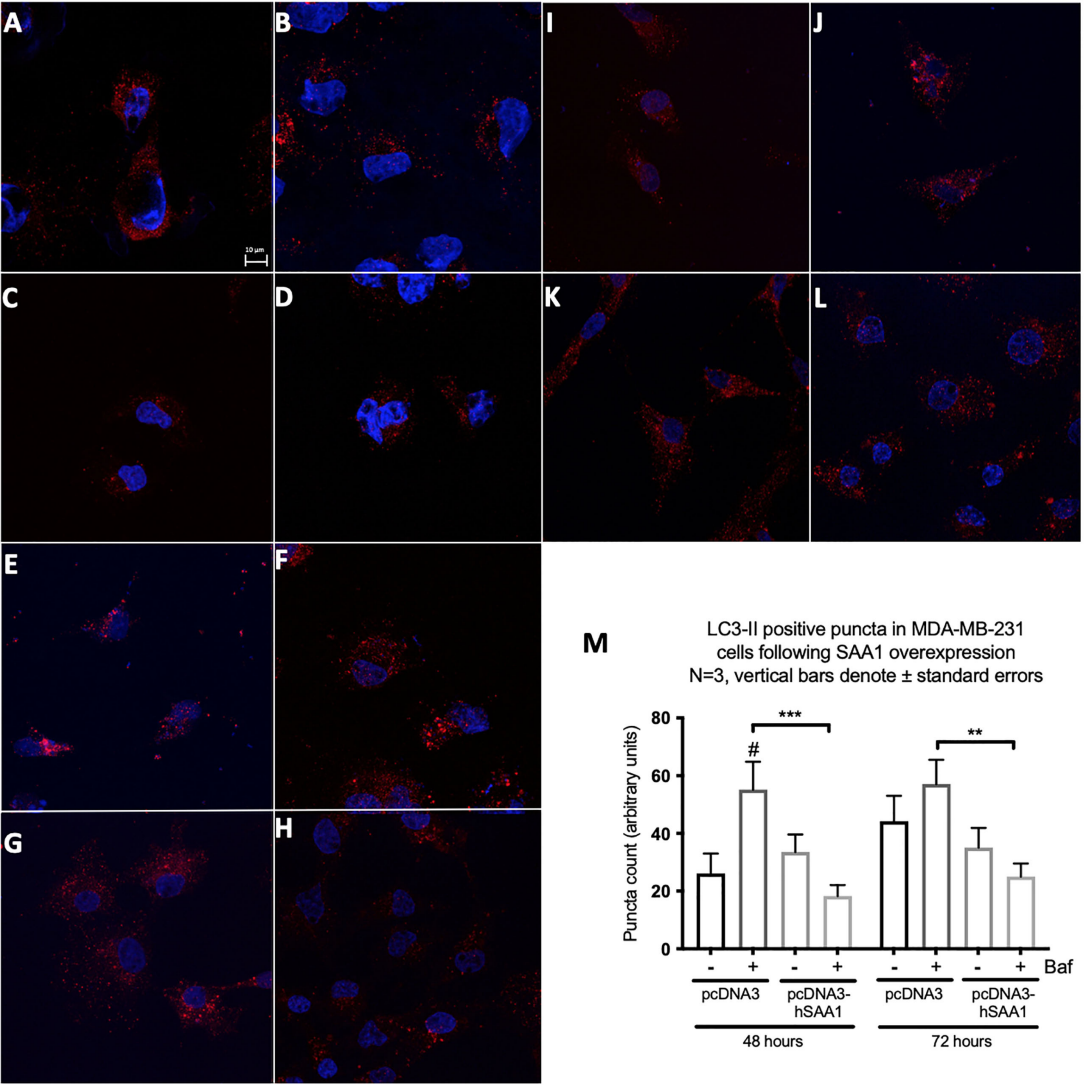
The expression of the autophagy marker, LC3-II, following SAA overexpression in the MDA-MB-231 cell line indicated by immunofluorescence and represented as mean fluorescent intensity. **(A)** Control. **(B)** 400 nM Bafilomycin for 4 hours. **(C)** 1% FBS for 8 hours. **(D)** 400 nM Bafilomycin with 1% FBS. **(E)** pcDNA for 48 hours. **(F)** pcDNA with Bafilomycin for 48 hours. **(G)** SAA overexpression for 48 hours. **(H)** SAA overexpression with Bafilomycin for 48 hours. **(I)** pcDNA3 for 72 hours. **(J)** pcDNA3 with Bafilomycin for 72 hours. **(K)** SAA overexpression for 72 hours. **(L)** SAA overexpression with Bafilomycin for 72 hours. **(M)** Mean fluorescent intensity of LC3-II following SAA overexpression for 48 and 72 hours. ***p < 0.001, # Indicates p < 0.05 when comparing a group with its respective Baf treated group. **p < 0.01.

**Figure 8:**
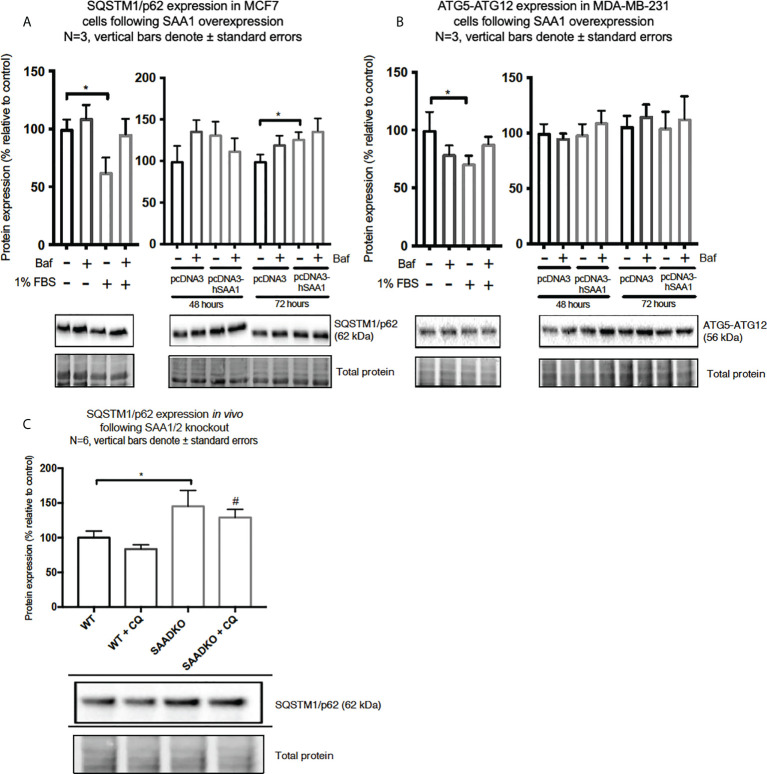
The expression of the autophagy markers, SQSTM1/p62 and ATG5-ATG12, following SAA1 overexpression in human breast cancer cells, or in tumors from WT and SAADKO mice, as indicated by western blot analysis. **(A)** SQSTM1/p62 expression following SAA overexpression for 48 and 72 hours in MCF7 cells. **(B)** ATG7-ATG12 expression following SAA overexpression for 48 and 72 hours in MDA-MB-231 cells. **(C)** SQSTM1/p62 expression *in vivo*. *p<0.05, # indicates p<0.05 when comparing the WT+CQ and SAADKO+CQ groups.

Western blot and immunocytochemistry experiments were performed to assess whether SAA regulates autophagy *in vitro* and *in vivo* (**Figures 5**–**8**). SAA1 overexpression in the MCF7 cell line downregulated autophagy following 48 and 72 hours as indicated by immunofluorescence (**Figure 6**), while western blot analyses showed that autophagy was downregulated following SAA1 overexpression for 72 hours (**Figure 5A**). Autophagy was significantly downregulated following SAA1 overexpression in the MDA-MB-231 cell line for 72 hours indicated by western blot analysis (**Figure 5B**). Furthermore, immunofluorescence results showed that the overexpression of SAA1 downregulated autophagy following 48 and 72 hours in the MDA-MB-231 (**Figure 7**). Knockout of SAA1/2 *in vivo* resulted in a significant increase in the accumulation of LC3-II positive autophagosomes in tumors when compared to the WT control (**Figure 5C**).

The expression of the cargo recruiter, SQSTM1/p62, was assessed to determine whether SAA influences autophagy in breast cancer cells (**Figures 8A–C**). The expression of SQSTM1/p62 was significantly increased following 72-hour SAA1 overexpression in the MCF7 cells (**Figure 8A**). However, no expression of SQSTM1/p62 was detected for the MDA-MB-231 cell line, therefore western blot experiments was performed to assess the autophagy machinery component, conjugated ATG5-ATG12 (**Figure 8B**). No significant changes were observed for ATG5-ATG12 in the MDA-MB-231 cell line. Knockout of SAA1/2 *in vivo* resulted in a significant increase in the accumulation of LC3-II positive autophagosomes in tumors when compared to the WT control (**Figure 8C**). Furthermore, the knockout of SAA1/2 resulted in a significant increase in the cargo recruiter, SQSTM1/p62, in tumors when compared to the WT control. Additionally, a significant increase in SQSTM1/p62 levels were observed when comparing the CQ treated WT and SAADKO tumor groups.

### SAA promotes proliferation *in vitro*

**Figure 9:**
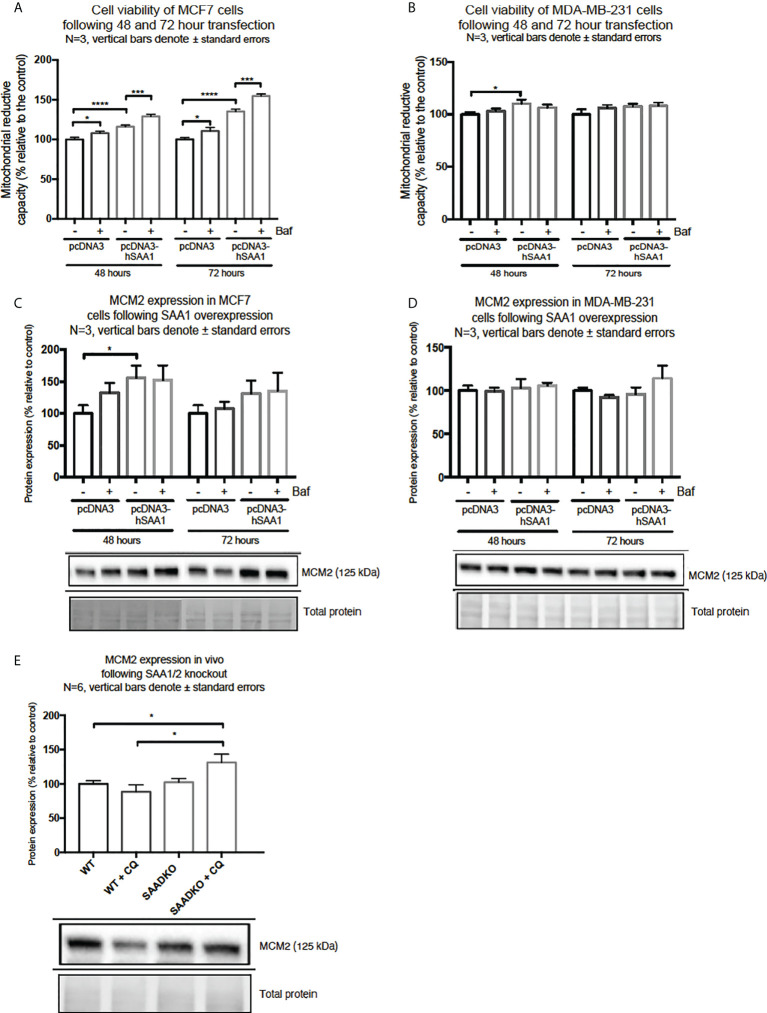
Cell viability and expression of the proliferation marker, MCM2, following SAA1 overexpression in human breast cancer cells, or in tumors from WT and SAADKO mice, as indicated by western blot analysis. **(A)** Cell viability following SAA overexpression for 48 and 72 hours in MCF7 cells. **(B)** Cell viability expression following SAA overexpression for 48 and 72 hours in MDA-MB-231 cells. **(C)** MCM2 expression following SAA overexpression for 48 and 72 hours in MCF7 cells. **(D)** MCM2 expression following SAA overexpression for 48 and 72 hours in MDA-MB-231 cells. **(E)** MCM2 expression *in vivo*. *p<0.05, ***p<0.001, ****p<0.0001.

SAA1 overexpression increased cell viability in MCF7 cells following 48 hour and 72 hours (**Figure 9A**). Furthermore, autophagy inhibition significantly increased cell viability in the MCF7 cell line in the 48-hour control Baf-treated group and the 72-hour control Baf-treated group when compared to their respective controls (**Figure 9A**). When compared to the control groups, SAA1 overexpression increased cell viability in MDA-MB-231 cells following 48-hour transfections (**Figure 9B**).

Next, we investigated the expression of the proliferation marker, MCM2 (**Figures 9C–E**). SAA1 overexpression resulted in a significant increase in the proliferation marker MCM2 following 48-hour transfections in the MCF7 cell line (**Figure 9C**). Furthermore, the expression of MCM2 was significantly upregulated when autophagy was inhibited in the control and SAA1 overexpression groups in the MCF7 cell line. For the MDA-MB-231 cell line, no significant changes in MCM2 expression were observed (**Figure 9D**). Lastly, the proliferation marker MCM2 did not significantly change following double knock out of SAA1/2 *in vivo*, however, the inhibition of autophagy with CQ significantly increased the expression of MCM2 in the SAADKO group when compared to the untreated WT and SAADKO tumor groups **(** **Figure 9E**).

### Double knockout of *Saa1* and *Saa2* protects against apoptosis in an autophagy-dependent manner *in vivo*

**Figure 10:**
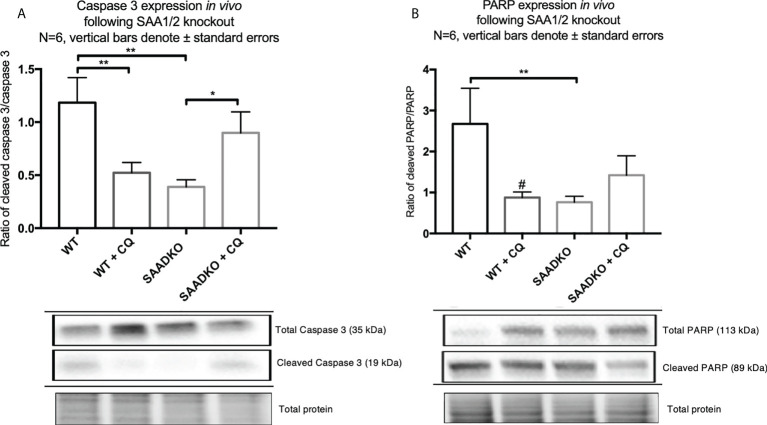
The expression of the apoptosis markers, caspase 3 and PARP, following double knockout of SAA1/2 overexpression indicated by western blot analysis. **(A)** Cleaved over total caspase 3 expression following double knockout of SAA1/2 *in vivo*. **(B)** Cleaved over total PARP expression following double knockout of SAA1/2 *in vivo*. *p<0.05, **p<0.01. # Indicates p<0.05 when comparing the WT and WT+CQ groups.

To assess whether double knockout of SAA1/2 can regulate apoptosis, we measured the expression levels of cleaved and total caspase 3 as well as cleaved and total PARP (**Figures 10A, B**). The ratio of cleaved caspase 3 to total caspase 3 was significantly decreased in the SAADKO group when compared to the WT control group, indicating that apoptosis was inhibited, which has been confirmed by other members of our group. However, the inhibition of autophagy with chloroquine significantly increased the cleaved caspase 3 to total caspase 3 ratio in the SAADKO group when compared to the untreated SAADKO group. Interestingly, the ratio of cleaved caspase 3 to total caspase 3 in the SAADKO with CQ group is increased to the same level of expression as the WT control (**Figure 10A**). The ratio of cleaved PARP to total PARP was significantly decreased in the SAADKO group when compared to the WT control group, indicating that apoptosis was inhibited (**Figure 10B**).

### Double knockout of *Saa1* and *Saa2* mediates necrosis

**Figure 11:**
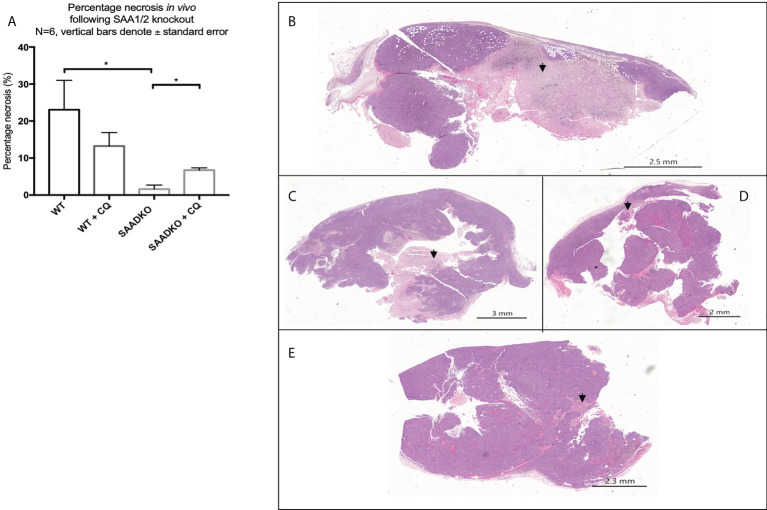
**(A)** The percentage necrosis following double knockout of SAA1/2 overexpression indicated by hematoxylin and eosin staining. Representative images for the Hematoxylin and Eosin staining to indicate necrosis. **(B)** Wild Type. **(C)** Wild type with chloroquine. **(D)** Double knockout of SAA1/2. **(E)** Double knockout of SAA1/2 with chloroquine. Arrows indicate areas of necrosis. *p<0.05.

The analysis of the tumor histology with hematoxylin and eosin stains showed that WT tumors had larger regions of tumor necrosis when compared to the SAADKO groups. Subsequently, SAADKO tumors showed larger areas of viable tissue (**Figures 11A–E**).

### Double knockout of *Saa1* and *Saa2* alters the cytokine profile *in vivo*

**Figure 12:**
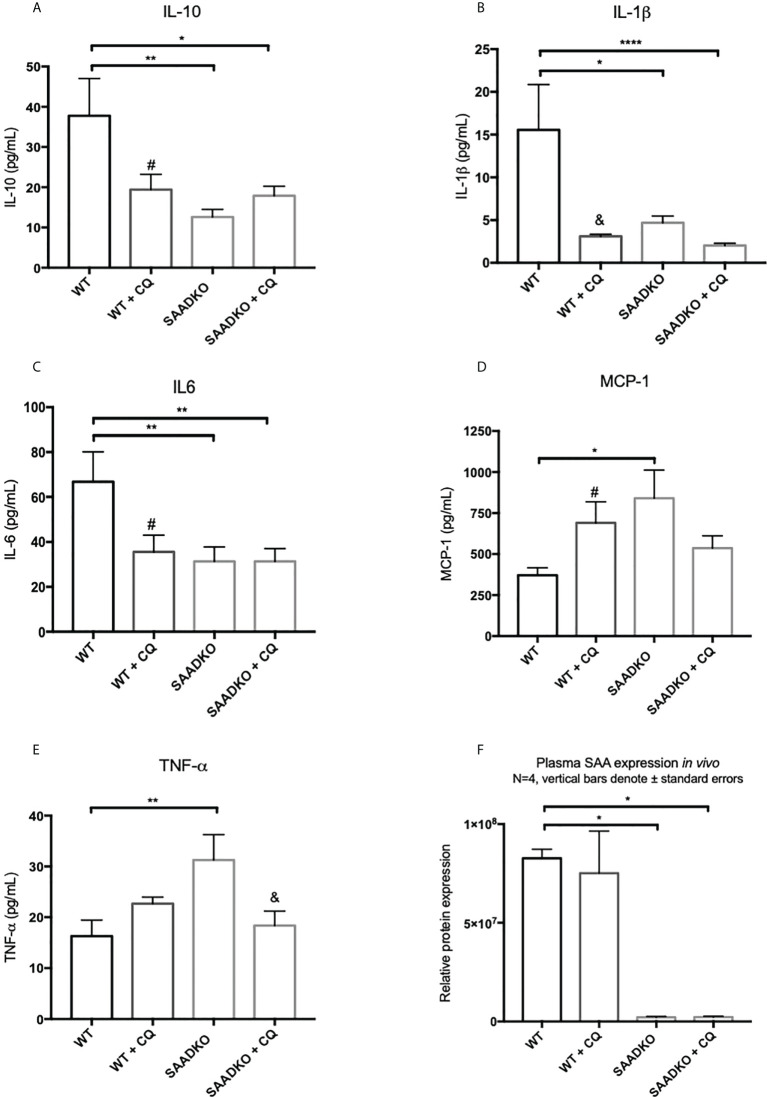
The cytokine profile of WT and SAADKO tumors. **(A)** Plasma levels of IL-10. **(B)** Plasma levels of IL-1β. **(C)** Plasma levels of IL-6. **(D)** Plasma levels of MCP-1. **(E)** Plasma levels of TNF-α. **(F)** Plasma levels SAA. *p<0.05, **p<0.01, ****p<0.0001. # Indicates p<0.05 when comparing a group with its respective chloroquine treated group. & Indicates p<0.01 when comparing a group with its respective chloroquine treated group.

Next, we assessed whether the double knockout of SAA1/2 alters the expression of pro-inflammatory and anti-inflammatory cytokines *in vivo* (**Figures 12A–F**). The levels of anti-inflammatory, IL-10, and the pro-inflammatory, IL-1β and IL-6, was significantly decreased in the WT group following autophagy inhibition, in the SAADKO group and in the SAADKO group following autophagy inhibition when compared to the WT control. In contrast, the knockout of SAA1/2 significantly increased the blood plasma levels of the pro-inflammatory markers, MCP-1 and TNF-α when compared to the WT control. Autophagy inhibition in the WT group significantly increased plasma MCP-1 levels when compared to the WT control, while autophagy inhibition in the SAADKO group significantly decreased plasma MCP-1 levels in the SAADKO group when compared to untreated SAADKO group. Autophagy inhibition also significantly decreased TNF-α levels in the SAADKO group when compared to the untreated SAADKO group.

## Discussion

### In vitro

In recent years, SAA has emerged as an important contributor to tumorigenesis, and is currently considered a clinical biomarker for breast cancer (9, 26). However, the exact mechanisms of action by which SAA promotes tumorigenesis still remain unclear. SAA has been reported to promote the activation of the mitogen activated protein kinases (MAPK), p38, ERK1/2 and JNK, as well as the phosphatidylinositol-3-kinase (PI3K) pathway component, Akt, in non-transformed cells such leukocytes, endothelial cells and adipocytes. These were mediated through the binding of SAA to various receptors, such as the formyl peptide receptor like 1 (FPRL-1), scavenger receptor class B type 1 (SR-BI), receptor for advanced glycation end products (RAGE) and toll-like receptors (TLR) 2/4 (12–19).

The activation of the p38, ERK1/2 and Akt signaling pathways play an important role in linking cellular responses and inflammatory stimuli, including SAA (27). Interestingly, the overexpression of SAA1 resulted in decreased Akt activation in the estrogen and progesterone receptor positive breast cancer cell line (MCF7) (**Figure 4A**). Furthermore, the overexpression of SAA1 with the inhibition of autophagy resulted in increased p38 activation (**Figure 2B**). The p38 MAPK can be activated by various factors such stress and inflammatory signals, such as SAA. The nature and strength of the stimulus determines the activation of p38, which can include the stimulation with SAA1 and concurrent autophagy inhibition (28). In addition, in line with previous literature (29, 30), the overexpression of SAA1 increased the activation of ERK in the triple-negative breast cancer cell line (**Figure 3B**). ERK is activated through Ras/Raf/MEK signaling cascades, where Ras activates the kinase activity of Raf, which activates MEK 1/2 through phosphorylation, which is subsequently activates ERK 1/2. These results indicate that the SAA-dependent activation or inhibition of these signaling pathways may be cell-type and context dependent. As such, tumors bearing the oncogenic Ras transformation is dependent on autophagy for metabolic fitness. Therefore, the role of autophagy induced by Ras depends strongly on the type of stimulus (31). More importantly, these signaling pathways are well known regulators of downstream processes, such as proliferation, autophagy, apoptosis and necrosis (32–34).

A functional relationship between SAA and autophagy has not yet been investigated, however we found that the overexpression of SAA1 downregulated autophagy *in vitro* (**Figures 5A, B**, **6**, **7**). It is also well established that Akt is a strong inhibitor of downstream autophagy, while p38 and ERK1/2 can either induce or inhibit autophagy based on the cellular context (35–37). The decreased activation of the autophagy inhibitor, Akt, in the MCF7 cell line (**Figure 4A**) could indicate that autophagy was downregulated by SAA1 in an Akt-independent manner, however further investigation is needed to confirm this. Furthermore, it has been reported that the ERK1/2 signaling pathway can act to either induce or inhibit autophagy, where this study reported that SAA1 can activate ERK1/2 as an inhibitor of autophagy in triple negative breast cancer cells (**Figures 3B**, **5B**). It is clear from literature that basal autophagy is an important mediator of homeostasis (38, 39). The activation of the PI3K/Akt signaling pathway inhibits autophagy through direct activation of mTOR *via* the phosphorylation of the mTORC1 subunit at S2448, or indirectly through the inhibition of the TSC1-TSC2 complex by phosphorylating the TSC2 subunit, destabilizing the interaction between TSC1 and TSC2 (40). Furthermore, ERK1/2 activation can induce the dissociation of the beclin-1 and Bcl-2, enabling autophagy induction or ERK-dependent phosphorylation of TSC2 leading to the dissociation of the TSC1-TSC2 complex, which can promote the inhibition of autophagy (41, 42). However, it may also be true that SAA regulates other signaling mechanisms, such as JAK/STAT3, that can regulate autophagy in cancer cells (43).

We have further also investigated whether SAA can regulate the cargo recruiter, SQSTM1/p62. The accumulation of SQSTM1/p62 is indicative of defective autophagy where SQSTM1/p62 and its tagged cargo cannot be degraded. In line with this, the expression of SQSTM1/p62 was increased in the MCF7 cell line when autophagy was downregulated by SAA1 (**Figure 8A**). Aberrant SQSTM1/p62 overexpression is implicated in breast cancer progression (44–46). Guo and Pei (47) reported that high expression of SQSTM1/p62 and low expression of Beclin1 and LC3-II/LC3-I in MDA-MB-231 cells lead to autophagy and apoptosis defects, accelerating breast cancer progression. SQSTM1/p62 also plays an important role at the crossroads of autophagy, apoptosis, and cancer (48, 49). Furthermore, Yu and co-authors (50) reported that the accumulation of SQSTM1/p62 in tumor cells *in vitro* was characterized by cell cycle initiation, which resulted in an increase in MCM2 expression, and enhanced proliferative ability, which is supported by our findings. Therefore, the inhibition of autophagy provides cancer cells with the means to sustain increased levels of proliferation and cell growth (34). Moreover, the overexpression of SAA1 and the resulting inhibition of autophagy promoted proliferation in the two breast cancer cell lines, evident in our results for the DNA replication licensing factor, MCM2 (**Figure 9C**), and cell viability (**Figures 9A, B**) as an indication of proliferation.

### In vivo

The *in vivo* findings indicate that the double knockout of SAA1/2 resulted in decreased activation of Akt (**Figure 4C**), which is consistent with previous literature (29, 30). We found that the double knockout of SAA1/2 promoted autophagy induction *in vivo* (**Figure 5C**), which is in line with our findings for Akt activation. Akt is a strong downstream inhibitor of autophagy, and here the decreased activation of Akt in the double knockout of SAA1/2 group correlates with increased autophagy. Furthermore, the *in vivo* results showed that SQSTM1/p62 is upregulated in SAADKO tumors, indicating that both autophagy and cargo recruitment is increased (**Figure 8C**). As such, SQSTM1/p62 is also a notoriously multifunctional protein, and in its role as an adapter protein, it contains a range of motifs which enable its interaction with various selective signal transduction pathways involved in cell proliferation and survival/death through regulation of transcription factor activities (51). In addition, homeostatic regulation may affect SQSTM1/p62 levels independent of autophagy.

Autophagy can also regulate apoptosis and necrosis (52). When cancer cells have deficient apoptosis, autophagy acts to sustain cell viability (22). The *in vivo* findings indicated that SAA1/2 knockout resulted in apoptosis resistance (**Figures 10A, B**), which is mediated through the induction of autophagy, where treatment of the knockout tumors with the autophagy inhibitor chloroquine increased apoptosis levels to that of the WT control, evident in our findings for caspase 3. As such, the time duration of the chloroquine treatment is optimal to detect changes in autophagy but not full completion of apoptosis, evident in our findings for PARP (**Figures 10A, B**). We also found that autophagy inhibition in WT tumors elicits a pro-tumorigenic response as it promotes apoptosis resistance, evident in the decreased levels of cleaved caspase-3 and PARP (**Figures 10A, B**). In contrast, tumor cells may be more prone to cellular stress in the absence of SAA1/2 and may upregulate autophagy as a cell protective response for survival. Furthermore, the double knockout of SAA1/2 tumors showed reduced levels of necrosis when compared to the WT controls (**Figures 11A–E**). The inhibition of autophagy in the SAADKO group significantly increased the percentage necrosis when compared to the untreated SAADKO group (**Figures 11A–E**). However, the assessment of other necrosis markers is required to validate these findings. In line with this, Degenhardt and co-authors (22) reported that autophagy deficiency promotes tumor necrosis, which supports our findings that autophagy, as a result of SAA deficiency, inhibits tumor necrosis *in vivo*. However, the decrease in necrosis was not entirely dependent on autophagy regulation alone and was partially facilitated by the knockout of SAA1/2, which also resulted in an altered cytokine profile.

Tissue necrosis stimulates a potent inflammatory response, while a fine balance exists between autophagy and inflammation, where autophagy can regulate inflammatory processes and inflammation can in turn regulate autophagy pathways (53–56). This is supported by our findings that SAADKO tumors are characterized by a smaller percentage of necrotic tumor tissue than the WT controls. As such, the levels of the pro-inflammatory IL-1β and IL-6 was significantly decreased in the SAADKO group when compared to the WT control (**Figures 12B, C**). However, it is also evident from our study that the expression of the interleukins is finely regulated by both knockout of SAA1/2 and autophagy inhibition in breast tumors. Other studies have reported that autophagy reduced the secretion of IL-1β *in vivo* and *in vitro* (57, 58). However, the inhibition of autophagy with 3-MA increased the expression of IL-1β and Il-6 (59). The inhibition of autophagy resulted in a similar decreased in pro-inflammatory cytokine levels than that of the SAADKO group, which displayed elevated levels of autophagy. Furthermore, the levels of the anti-inflammatory interleukin, IL-10, was also significantly lower in the SAADKO group and when autophagy was inhibited in both the WT and SAADKO groups (**Figure 12A**). In contrast, an interesting finding was that the levels of pro-inflammatory TNF-α and MCP-1 was increased in SAADKO tumors (**Figures 12D, E**). TNF-α can upregulate the expression of autophagy genes MAP1LC3B and BECN1, coding for LC3 and beclin-1 respectively, while also inhibiting Akt activation, while it can also induce autophagy through the ERK1/2 pathway (53, 54). Furthermore, Liu and co-authors (60) reported that autophagy can induce TNF-α production. Autophagy inhibition, similar to the results of cleaved caspase-3, increased MCP-1 levels in the WT group, while autophagy inhibition decreased MCP-1 levels in the SAADKO group. Therefore, the altered cytokine profile as a result of SAA1/2 knockout can, in turn, promote autophagy.

**Figure 13:**
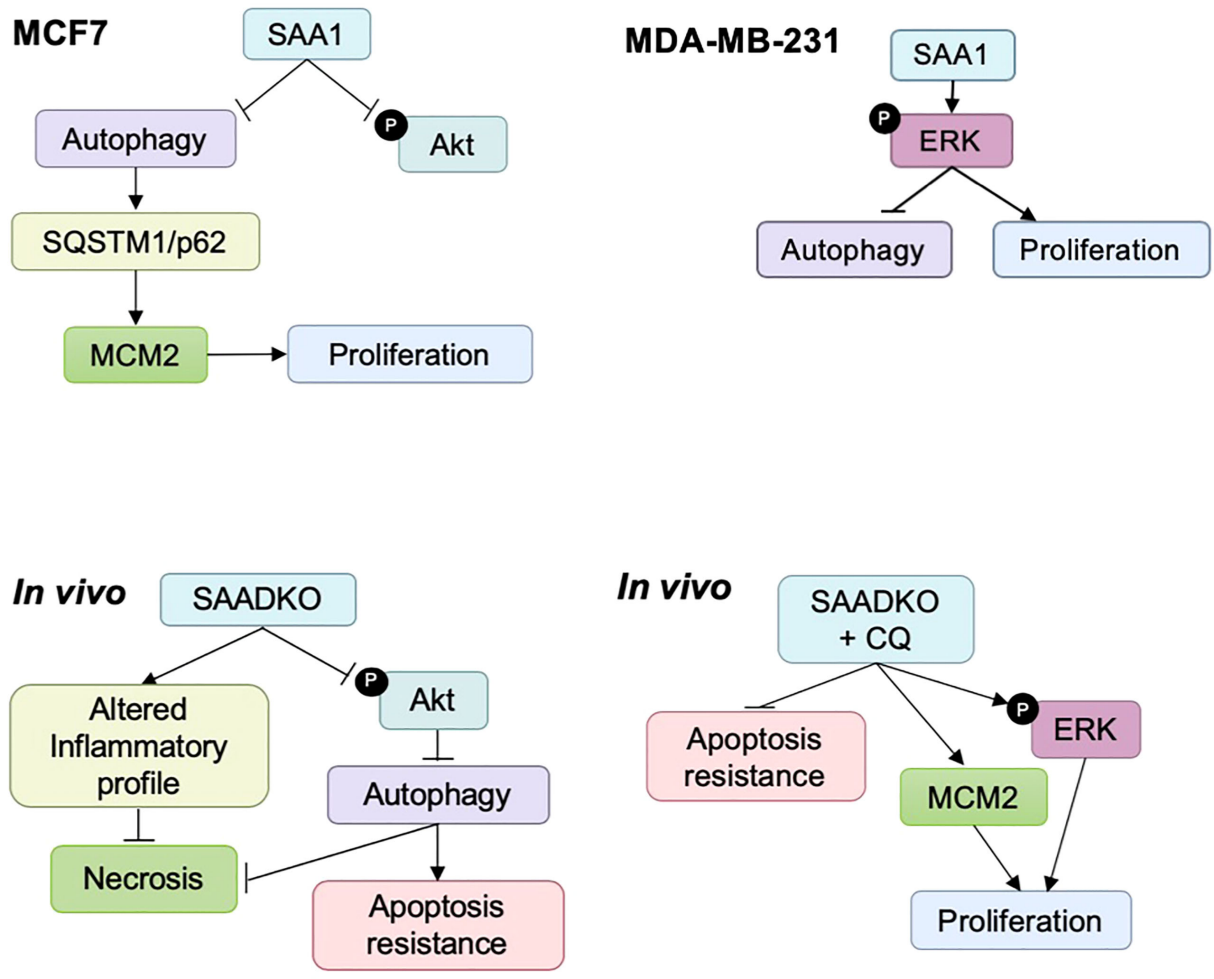
Summary diagrams of the *in vitro* and *in vivo* results.

We reported various differences and similarities between the MCF7 and MDA-MB-231 cell lines in response to SAA overexpression. SAA downregulated the activation of Akt in the MCF7 cell line and promoted ERK activation in the MDA-MB-231 cell line, while downregulating autophagy and promoting proliferation in both cell lines. Following the double knockout of SAA1/2, the activation of Akt was decreased, autophagy was induced, while promoting apoptosis resistance and decreased necrosis. Finally, the double knockout of SAA1/2 contributed to an altered cytokine profile *in vivo* by reducing the circulating levels of IL-1β, IL-6 and IL-10 and increasing TNF-α and MCP-1. It is well established that cancer is closely associated with inflammation, including SAA, evident in the high rates of proliferation and elevated levels of pro-inflammatory cytokines present in the TME. Furthermore, from our results it is clear that the intracellular degradation process, autophagy, remains central in tumorigenesis depending on the cellular context. In conclusion, it is evident that some SAA-dependent mechanisms of tumorigenesis still remains unclear such as genetic perturbations of autophagy, however the findings of this study clearly indicate the significance of the role of SAA in models of breast cancer (**Figure 13**).

## Data availability statement

The original contributions presented in the study are included in the article/**Supplementary Material**. Further inquiries can be directed to the corresponding author.

## Author contributions

MdP was responsible for the conceptualization, methodology, validation, formal analysis, investigation, writing the original draft, review and editing, visualization and project administration. TD was responsible for conceptualization, methodology, review and editing and supervision. DWO was responsible for methodology, review and editing. WJSdV was responsible for review and editing. AME was responsible for the conceptualization, resources, review and editing and supervision. All authors contributed to the article and approved the submitted version.

## Funding

Funding was obtained from the National Research Foundation of South Africa (NRF) (grant number 118566), the Cancer Association of South Africa (CANSA) and the South African Medical Research Council (SAMRC).

## Conflict of interest

The authors declare that the research was conducted in the absence of any commercial or financial relationships that could be construed as a potential conflict of interest.

## Publisher’s note

All claims expressed in this article are solely those of the authors and do not necessarily represent those of their affiliated organizations, or those of the publisher, the editors and the reviewers. Any product that may be evaluated in this article, or claim that may be made by its manufacturer, is not guaranteed or endorsed by the publisher.

## Supplementary material

Supplementary Figure 1
**(A)** The tumour growth of WT and SAADKO tumours over time. **(B)** The Kaplan-Meier survival analysis showing the cumulative proportion of surviving mice throughout the study.
