# Supplementary figures and images for: A functional role for Serum Amyloid A in the molecular regulation of autophagy in breast cancer
Source: Front Oncol. 2022 Sep 30;12:1000925. doi: 10.3389/fonc.2022.1000925 (PMC9562844; doi:10.3389/fonc.2022.1000925)

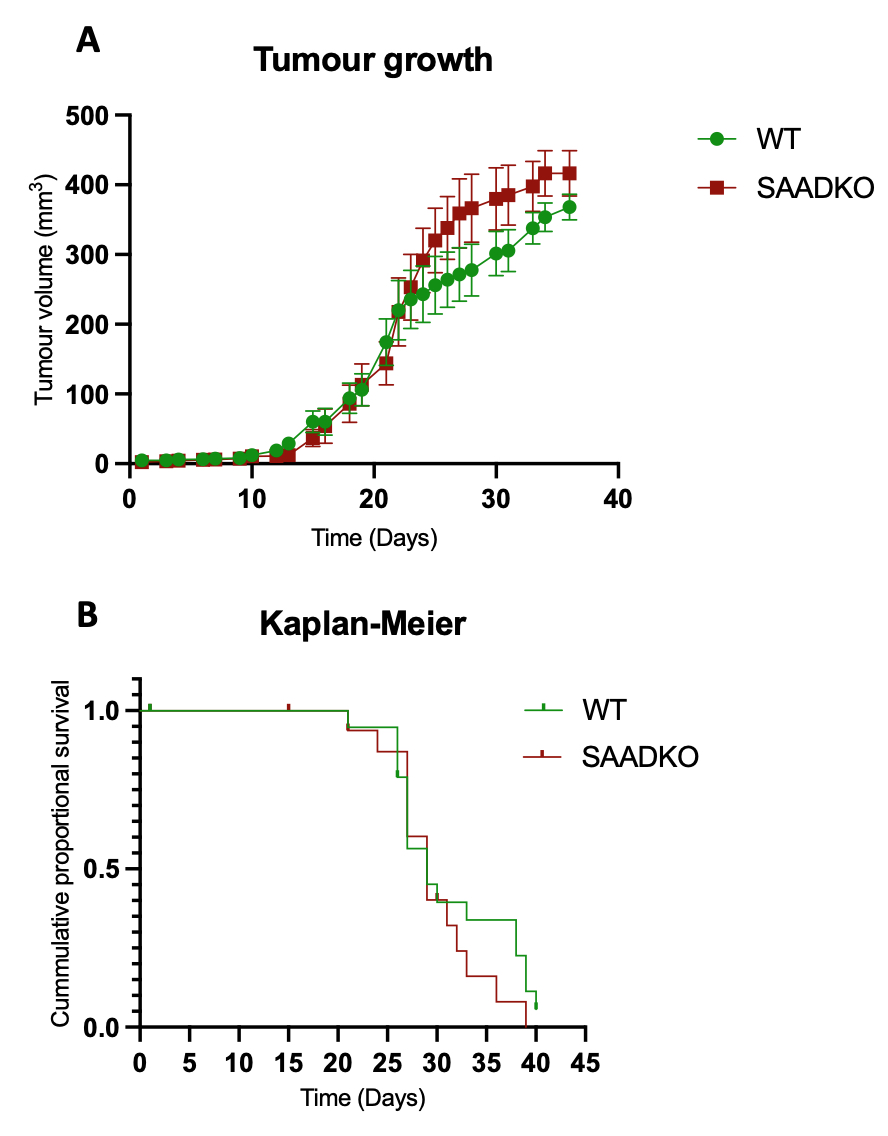

Supplement: Supplementary Figure 1 — (A) The tumour growth of WT and SAADKO tumours over time. (B) The Kaplan-Meier survival analysis showing the cumulative proportion of surviving mice throughout the study. [file Image_1.jpeg]
